# The impact of palliative care contact on the use of hospital resources at the end of life for brain tumor patients; a nationwide register-based cohort study

**DOI:** 10.1007/s11060-025-04939-9

**Published:** 2025-01-20

**Authors:** Nelli-Sofia Nåhls, Anu Anttonen, Mikko Nuutinen, Tiina Saarto, Timo Carpén

**Affiliations:** 1 Department of Oncology, Vaasa Central Hospital, The Wellbeing Services County of Ostrobothnia, Vaasa, Finland; 2 Department of Oncology, Comprehensive Cancer Centre, University of Helsinki, Helsinki, Finland; 3 Department of Radiotherapy, Comprehensive Cancer Center, Helsinki University Hospital, Helsinki, Finland; 4 Nordic Healthcare Group, Helsinki, Finland; 5 Department of Palliative Care, Comprehensive Cancer Center, Helsinki University Hospital, and Faculty of Medicine, Helsinki University, Helsinki, Finland

**Keywords:** Specialist palliative care, Brain tumor, Emergency department, Healthcare utilization, End of life care

## Abstract

**Purpose:**

The aim of this nationwide retrospective cohort study was to evaluate the timing of the first specialist palliative care (SPC) contact and its impact on the use of hospital resources at the end of life in patients with brain tumors.

**Materials and methods:**

The analysis comprised 373 brain tumor patients who died during 2019 in Finland. Patients were divided into two groups according to the time of first SPC contact: early, i.e. first SPC contact more than 30 days before death, and late, i.e. no SPC contact or 30 days or less before death.

**Results:**

216 (58%) were male, with a mean age of 67 years (range 18–94). SPC contact was established for 102 (27%) patients and the median time of first SPC contact before death was 76 days. Patients with an early SPC contact had fewer outpatient clinic contacts (28% vs. 53%; p-value < 0.001) and fewer hospitalization (10% vs. 37%; p-value < 0.001) in secondary care compared with patients with late SPC contact. Early SPC contact had no impact on emergency department contacts. Patients with early SPC contact were more likely to die at long term care facility or in SPC wards instead of hospital (p-value < 0.001) compared to patients with late SPC contact (hospital deaths 51% vs. 80%, respectively).

**Conclusions:**

Early SPC contact reduced the burden on secondary care for brain tumor patients in the last months of life. Palliative care contact should be offered early to all brain tumor patients.

**Supplementary Information:**

The online version contains supplementary material available at 10.1007/s11060-025-04939-9.

## Introduction

In Finland, with a population of 5.5 million, more than 400 new cases of malignant glioma are diagnosed every year [1]. The most common adult brain tumor, glioblastoma, has a poor prognosis with a 5-year relative overall survival rate of 6.8% [2]. Because of a life-limiting disease, it is particularly important to identify and investigate the implementation of palliative care.

A large proportion of brain tumor patients have symptoms caused by a tumor mass (e.g. headaches, seizures, neurocognitive impairment) that require hospital services [3–4]. The complexity of symptoms makes palliative care for brain tumor patients challenging. Typically, symptoms increase towards the end of life (EOL), and the need for palliative care increases to control symptom burden and improve quality of life (QOL) [4, 5, 6].

Although the need for symptomatic care is undisputed, palliative care for brain tumor patients is limited worldwide [7, 8, 9]. Previous studies have suggested that brain tumor patients may benefit from early specialist palliative care (SPC) integration [10]. SPC also reduces the need for healthcare for brain tumor patients, including emergency department visits and hospitalizations [11]. Likewise, timely decision to terminate life prolonging anticancer treatments and concentration on symptom centered palliative care e.g. palliative care decision reduced emergency department visits and hospitalizations in tertiary hospital for brain tumor patients [12].

The aim of our nationwide retrospective cohort study was to investigate the timing of a SPC contact and its impact on acute hospital recourse use and place of death at EOL. To our knowledge, no previous nationwide data on the use of acute hospital resources for brain tumor patients at EOL has been reported.

## Materials and methods

### Cohort selection

The cohort consisted of 373 patients with malignant brain tumor who died in 2019. The patients were identified nationwide from the 2019 Causes of Death Register (Statistics Finland) using the International Classification of Disease (ICD-10) coding for Neoplasma malignum cerebri (C71.0-9). The total number of patients fulfilling the criteria was 375. Finally, 373 patients were included as two patients who died outside of Finland were excluded from the study.

### Data collection

Finnish healthcare authorities have established two comprehensive databases: the National Care Register and Kanta Services. The latter is a digital platform serving both social welfare and healthcare sectors. These standardized information systems are mandated by law, requiring participation from all healthcare providers, regardless of their public or private status.

Data on sociodemographic information, use of healthcare and social services and SPC services were obtained from these databases. The data were linked to a health service unit code list to identify the use of different services, including palliative care units. The data collection was carried out from the beginning of 2018 until the end of 2019.

### Utilization of health and social care services

In Finland, all residents have access to publicly funded healthcare, which includes a range of services from primary to tertiary care, as well as emergency and social care service. At the time of this study, primary health care was provided by municipalities, with general practitioners responsible for care including in primary care hospitals. Secondary and tertiary care represented specialized medical care. Secondary care was delivered through 20 hospital district hospitals, while tertiary care was provided by five university hospitals, each serving its designated catchment area.

To identify the use of various services, including palliative care units, we linked the collected data to a health service unit code list. This allowed us to classify and analyze the use of different types of care in different areas of health care.

Data on the use of health and social services was collected nationally; outpatient clinic contacts in primary, secondary and tertiary health care, hospitalizations in primary, secondary and tertiary health care, emergency department contacts including contacts to primary, secondary and tertiary health care, social care service utilization, home care service (which in Finland mainly refers to home-based assistance provided by a practical nurse, less often a doctor or nurse visit) and SPC service utilization.

For the purposes of this study, we combined secondary and tertiary care data and refer to it collectively as “secondary care” throughout the article.

### Palliative care contact

In Finland, palliative care services are divided into general and specialist level. In this study, we focused on SPC provided in both primary care and secondary care and defined it as a single group.

The SPC contact included a specialized palliative care outpatient clinic, a hospital at home (which is hospital-level care at home), a specialized inpatient ward or hospice, and palliative care inpatient consultations, all provided by a palliative care specialist. Each SPC visit or appointment and hospitalization (discharge) was equivalent to one contact.

In the study, patients were divided into groups according to the time of first SPC contact; (I) first SPC contact more than 30 days before death, (II) no contact or 30 days or less before death.

### Place of death

Place of death was determined from data in the 2019 Cause of Death Register and classified as home, long term care facility, or hospital (including primary health care and secondary hospitals). In addition, place of death was reported as SPC ward if the patient was under SPC care in a specialized palliative care ward at the time of death.

### Ethical statement

The study was performed with the Finnish Institute for Health and Welfare (THL) as part of the Project on Quality Information on Palliative Care and End-of-life Care. The study was approved by THL Dnr: 12,345,556. According to the Finnish legislation for research, no separate ethics committee approval was needed, as data used in the study consisted of deceased patients.

### Statistical analyses

All statistics were performed using IBM-SPSS version 29 (IBM Corp, Armonk, NY, USA). Descriptive statistics are reported as medians and ranges, numbers of incidences and percentages. The groups were divided according to the timing of the first SPC contact. Pearson’s chi-squared test and Fisher exact tests were used to compare categorical variables. Comparisons between hospital days in different groups were done using Mann-Whitney test as the distributions were not equal. A p-value of < 0.05 was considered statistically significant.

## Results

**Fig. 1:**
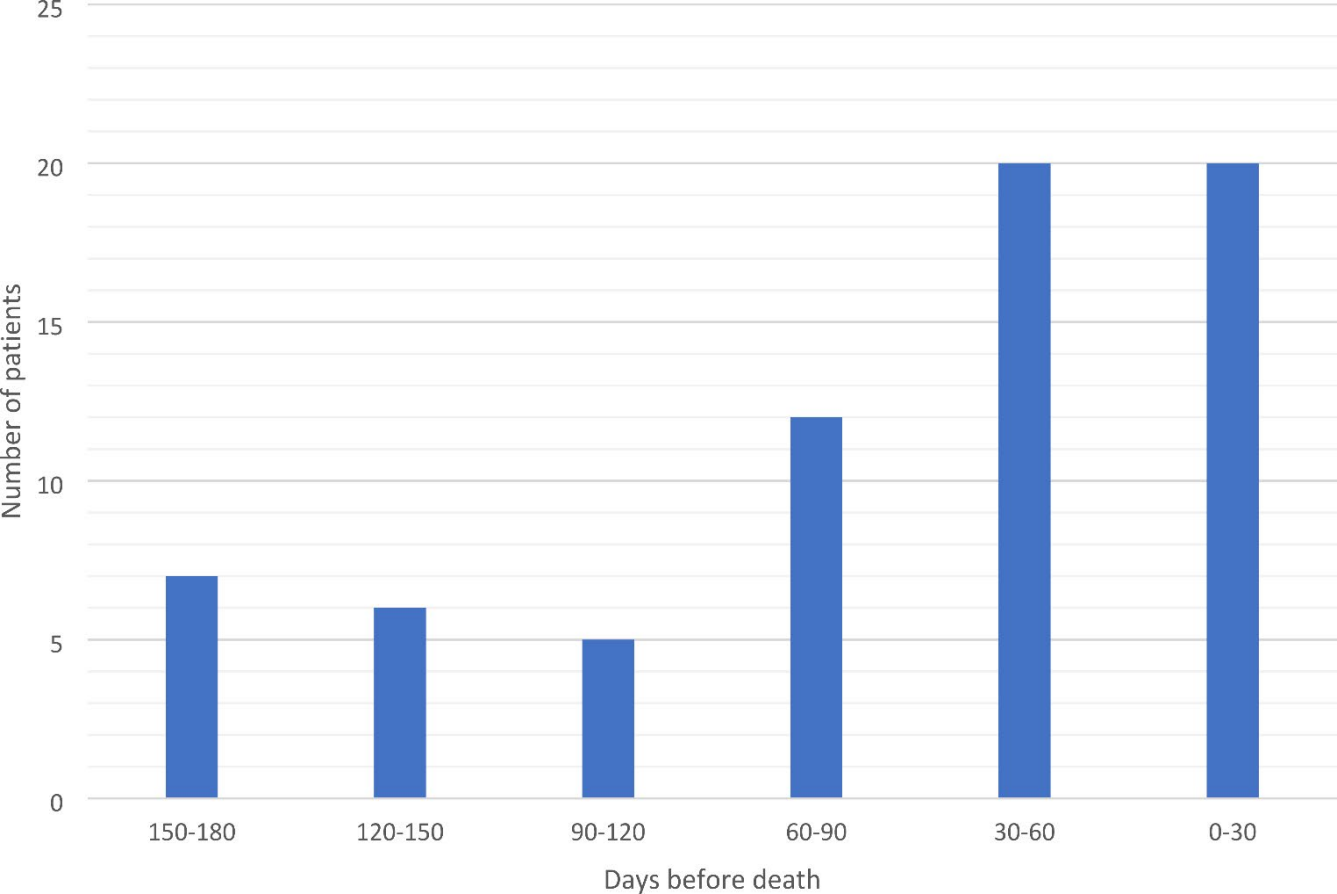
Timing of first SPC contact before death

Patient characteristics are shown in Table 1. The final cohort comprised 373 patients. Patients were divided into two groups according to first SPC contact; group (I) early SPC contact with first contact to SPC at least 31 days before death, and group (II) late or no SPC contact with no SPC contact or first contact 30 days or less before death. The mean age of the total population was 67 years at the time of death; 216 (58%) of the patients were male. SPC contact was established for 102 (27%) patients during their illness and for 81 (22%) patients more than 30 days before death. In the early SPC contact group (group I), the median time to first SPC contact was 138 days (range 33–708 days). Among those in group II who received SPC (*n* = 21), the median time to first contact was 17 days (range 1–27 days). It’s important to note that the majority of patients in group II (271 out of 292) had no SPC contact at all. The timing of the first SPC contact is shown in Fig. 1.

**Table 1:** Patient characteristics

Patients *N* = 373	Number (%)	Group I early SPC contact (*n* = 81)	Group II Late or no SPC contact (*n* = 292)	*P*-value
Age in years median (range)	70 (18–94)	70 (23–93)	70 (18–94)	0.605
Sex				0.818
Male	216 (58%)	46 (57%)	170 (58%)	
Female	157 (42%)	35 (43%)	122 (42%)	
ICD-10 Diagnosis code Z51.5 Palliative care	161 (43%)	55 (68%)	106 (36%)	< 0.001
Special palliative care (SPC) contact	102 (27%)	81 (100%)	21 (7%)	< 0.001
Median time from the first SPC contact to the death	76 days (0-708 days)	138 days (33–708 days)	17 days (0–27 days)	< 0.001

The use of hospital services and place of death within the last 30 days of life in group I and II are shown in Table 2.

**Table 2:** The impact of timing of first specialist palliative care (SPC) contact on the use of hospital services and place of death in the last 30 days of life

Patients *N* = 373	All	Group I Early SPC contact (*n* = 81)	Group II Late or no SPC contact (*n* = 292)	*P*-value
Emergency department contacts	142 (38%)	28 (35%)	114 (39%)	0.463
Outpatient clinic contacts				
Secondary care	179 (48%)	23 (28%)	156 (53%)	< 0.001
Primary health care	243 (65%)	48 (59%)	195 (67%)	0.209
Hospitalizations				
Hospitalizations all	262 (70%)	39 (48%)	223 (76%)	< 0.001
Secondary care	115 (31%)	8 (10%)	107 (37%)	< 0.001
Inpatient days (median)	7 days (range 1–30)	4 days (2–9 days)	7 days (range 1–30)	< 0.001
Primary health care	209 (56%)	35 (43%)	174 (60%)	0.009
Inpatient days (median)	26 days (range 1–30)	22 days (1–30 days)	27 days (range 1–30 days)	0.006
Specialist palliative care				
Palliative care outpatient unit	24 (6%)	13 (16%)	11 (4%)	< 0.001
Hospital at home	34 (9%)	27 (33%)	7 (2%)	< 0.001
Special palliative care ward	29 (8%)	22 (27%)	7 (2%)	< 0.001
Inpatient days (median)	18 days (range 1–30)	20 days (1–30 days)	9 days (1–19 days)	< 0.001
Social services	62 (17%)	21 (26%)	41 (14%)	0.011
Home care	128 (34%)	39 (48%)	89 (31%)	0.003
Place of death				< 0.001
Home	41 (11%)	8 (10%)	33 (11%)	0.717
Hospital	273 (73%)	41 (51%)	232 (80%)	< 0.001
Long term care facility	36 (10%)	15 (18%)	21 (7%)	0.002
Specialist palliative care ward	23 (6%)	17 (21%)	6 (2%)	< 0.001

### Use of hospital services

**Fig. 2:**
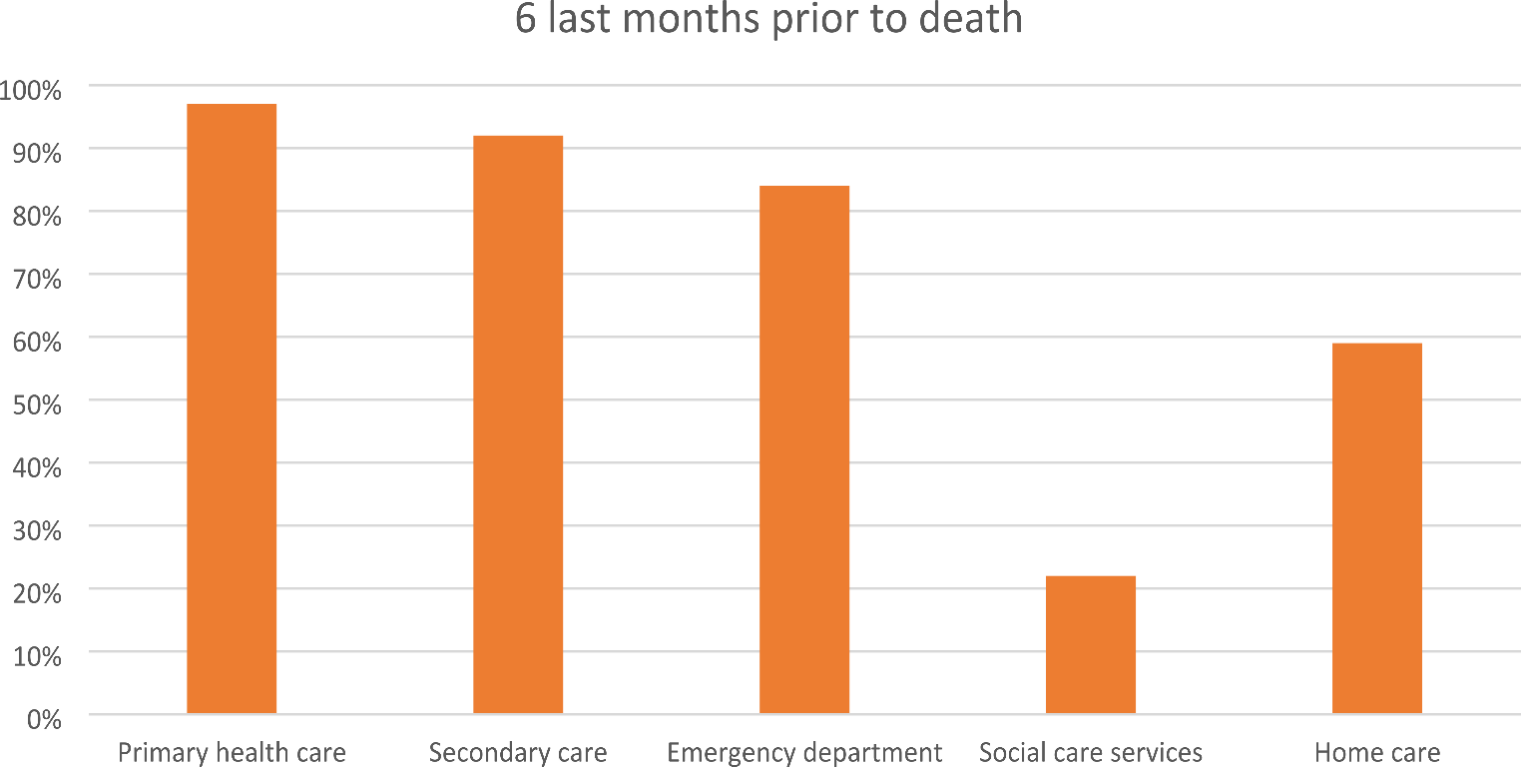
Percentage of patients who used specific health care or social care services 6 month before death

Brain tumor patients had a high use of hospital services in the last 6 months of life. Almost all patients had outpatient clinic and emergency department contacts. A large proportion were also hospitalized. Health care use in the last 6 months of life is shown in Fig. 2.

**Fig. 3:**
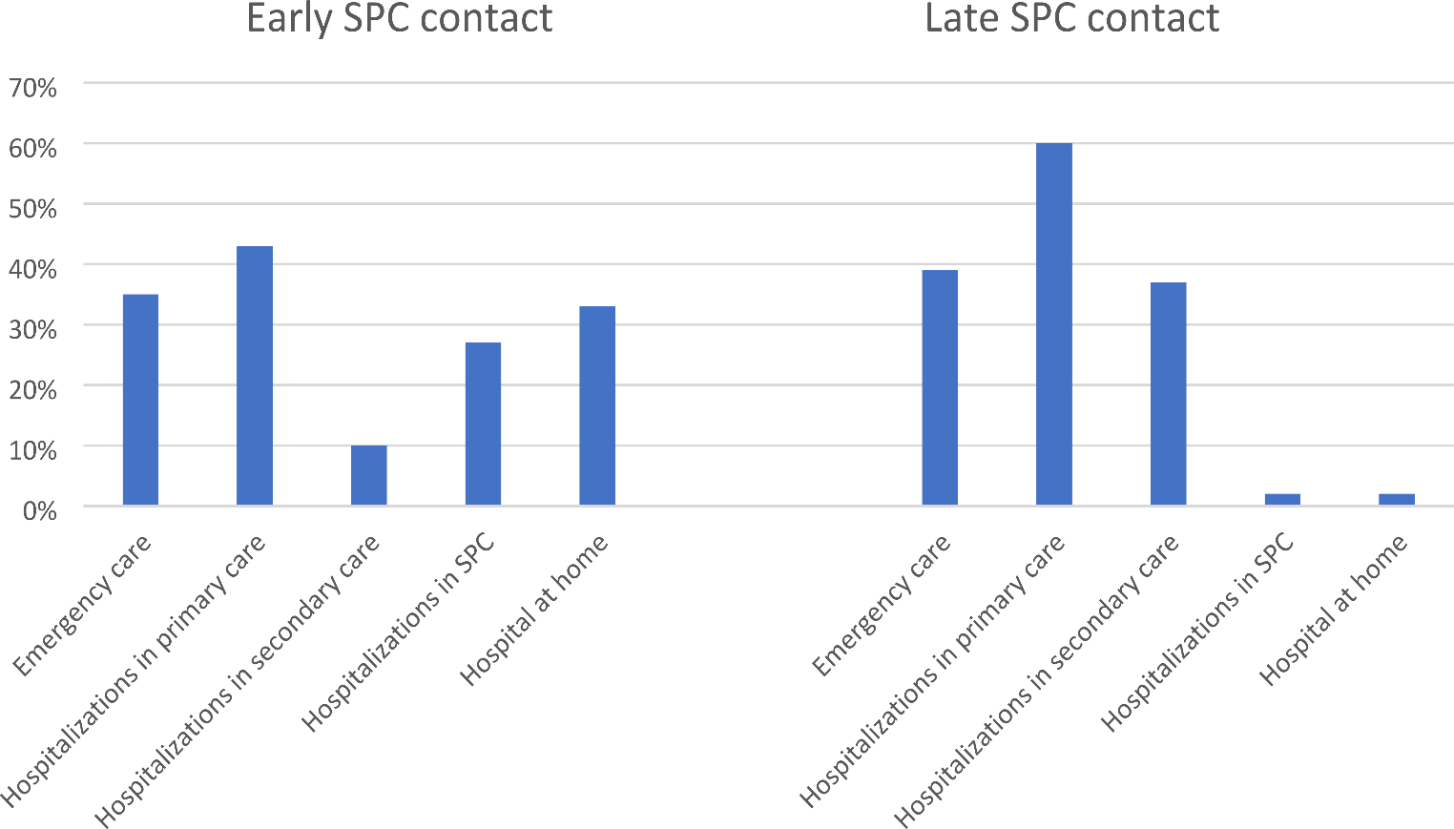
Emergency department contacts and hospitalizations in the last month of life according to the time of first specialist palliative care (SPC) contact

In the last month of life, 38% of patients had an emergency department contact in secondary care or/and primary health care. Emergency department contacts by time of first SPC are shown in Fig. 3. There was no difference between the groups in emergency department contacts.

In the last month of life, 31% of patients were hospitalized in a secondary care. Of them 20% (23 patients) patients were hospitalized in an oncological ward, none of them early SPC contact (p-value 0.009). More than half of patients (56%) were hospitalized in primary health care in the last month of life. Patients with an early (> 30 days before death) SPC contact had fewer hospitalizations in secondary hospitals (p-value < 0.001) and in primary health care hospitals (p-value 0.009). Hospitalizations by time of first SPC are shown in Fig. 3. The average length of hospital stay in the last 30 days of life at secondary care was 7 days (4 days in group I vs. 7 days in group II, p-value < 0.001) and in primary health care 26 days (22 days in group I vs. 27 days in group II, p-value 0.006).

Patients with an early (> 30 days before death) SPC contact had fewer contacts at secondary care outpatient clinic (28% vs. 53%, *p* < 0.001) during the last month of life. 31% (117 patients) of patients had an oncology outpatient clinic contact in the last month of life: 13% of the patients with early SPC contact vs. 36% with late or no SPC contact (*p* < 0.001). After the first SPC contact, 19 patients (5%) had an oncology outpatient clinic contact.

9% of the patients had hospital at home service in the last month of life, almost all (79%) of whom had an early first SPC. 34% (128) of patients had home care service in the last month of life.

### Place of death

Most patients (73%) died in hospital. EOL care at home was rare. Early SPC contact was associated with death at long term care facility or in SPC ward, these patients had 28% less deaths at the hospital (51% vs. 80%, respectively, p-value < 0.001) (Table 2). Patients who had a hospital at home in the last month of life were more likely to die at home, long term care or SPC ward than in hospital compared to patients who did not have (29% vs. 9% at home, 15% vs. 9% at long term care, 18% vs. 5% at SPC, and 38% vs. 77% at hospital, *p* < 0.001, respectively). Hospital at home did not reduce emergency department contacts (50% vs. 37%, *p* = 0.133), but it did decrease hospitalizations in secondary care (15% vs. 32%, *p* = 0.033), with no statistical effect on primary health care (44% vs. 57%, p-value 0.142).

## Discussion

A real-life nationwide data on EOL care pathways for brain tumor patients shows that early SPC contact reduced the burden on secondary care, both in terms of outpatient clinic contacts and hospitalizations. Early SPC contact also allowed a greater proportion of patients to die at long-term care, or SPC rather than in hospital compared to patients who did not have SPC contact or had it late (30 days or less before death).

Most brain tumors are not curable highlighting the importance of palliative care. Despite of the international recommendations [4], in our study, only 27% of patients had a SPC contact during their illness. However, contact with the SPC was made relatively early, on median 76 days before death. Our results were in line with other studies suggesting that referral to palliative care of brain tumor patients is limited. In a systematic review, Wu et al. [13] found that palliative care was low in patients with glioblastoma, documentation of advance care planning ranged from 4 to 55%, palliative referral was made in 39–40% of patients (palliative care consultation utilization rate reported as 34–36%) and hospice care in 66–76% of patients (utilization rates ranged from 38 to 86%). A similar result was obtained in a population-based retrospective study of 1784 glioblastoma patients, which also showed low rates of palliative care: only 34% of patients received palliative care and survival after palliative referral was similar to our study; 100 ± 160 days [14].

In our previous retrospective study, we found that early palliative care decision (e.g., the decision to terminate curative or life-prolonging anticancer treatments and focus on symptom-centered palliative care) reduced emergency department visits and inpatient stays in tertiary hospital for brain tumor patients [12]. A Swedish population-based registry study observed a decrease in emergency department visits among brain tumor patients who had received specialized palliative care in the last month of life [11], a similar finding in a heterogeneous group of cancer patients where a visit to a palliative outpatient clinic had a reducing effect on acute hospital resource use during the last 30 and 90 days before death [15]. However, in contrast to these findings, we were not able to repeat these findings. This unexpected result warrants further exploration. Several factors could potentially explain this discrepancy. Firstly, the complex and often unpredictable nature of neurological symptoms in brain tumor patients may necessitate emergency care regardless of SPC involvement. Secondly, the limited capacities of SPC services in Finland, such as 24/7 availability and the support from doctor during emergency hours could, at least partly, explain the inability to manage acute symptoms at home.

Our study found that early SPC contact reduced significantly contacts at secondary outpatient clinics and oncology outpatient clinics. In the last month of life, patients with early SPC contact were more likely to have a palliative outpatient clinic contact, compared to those with late/no contact. A small proportion of patients (5%) had an oncology outpatient clinic contact after the first SPC contact, representing early integrated palliative care. At the time of the study, this was not routine practice across the country.

A high proportion of patients in our cohort were hospitalized in the last month of life (31% in secondary hospital ward and 56% in a primary health care ward). This seemed to be prevented by early SPC contact as it was significantly associated with fewer hospitalizations in primary health care and in secondary care. In a single-center study of 385 glioblastoma patients 42.6% of patients were hospitalized in the last month of life, and 34% of these were admitted to intensive care [16]. In a retrospective cohort study of 5029 elderly glioblastoma patients, Arvold et al. found that 21% of patients were hospitalized for at least 30 cumulative days between diagnosis and death and 22% were hospitalized for at least one quarter of their remaining lifetime [17]. In another single-center retrospective cohort study 37% of glioblastoma patients were hospitalized for an average of nine days in the last month of life [18]. These results are in line with our study of secondary hospitalizations. In our study, the use of health and social care services was high in the last 6 months of life. The high number of hospitalizations in primary health care is partly due to the fact that there were few SPC wards or hospices available in Finland and therefore EOL care often took place in a primary health care ward.

A pilot program involving 848 brain tumor patients studied the impact of home care (including a neurologist visit at home, neurorehabilitation at home, psychological support for patients and their families, and nursing assistance), particularly at the EOL. 61% were treated at home until death, only 22% died in hospital. Patients who had home care had fewer hospital readmissions [19]. In our study, a similar service was hospital at home, and only 9% of patients received it in the last month of life. Patients who had a hospital at home were more likely to die in a home/long-term care/SPC setting (62% vs. 23%) than in hospital (38% vs. 77%). Hospital at home was not associated with emergency department contacts, but it did significantly decrease hospitalization in secondary care. A possible reason could be that by the time of the study, during the on-call hours, hospital at home nurses could not consult palliative care specialist, which led to contacts in the emergency department. Early SPC contact, and in particular hospital at home, increased the likelihood of death at home. The modest use of hospital at home is explained by the fact that it was not widely available, but early SPC contact improved access to hospital at home. Patients with early SPC contact were also more likely to use home care and social services in the last month of life.

Deaths at home were rare in our study population. Most study patients (73%) died in hospital. Compared to other studies, the rate of hospital deaths was higher. In a study by Koekkoek JA et al. comparing EOL care processes for high-grade glioma patients in three European countries, the Neatherlands, Austria and the United Kingdom, the majority of patients (60%) died at home, in hospital (41%) or in hospice care (41%, respectively [20]. According to a systematic review of patients with high-grade glioma the place of death varied depending on the study designs. Thus, comparing place of deaths across countries and studies is challenging [21].

The strengths of the study are a nationwide design, including both primary health care and secondary care outpatient clinic contacts, hospitalization, and emergency department contacts, which allowed a comprehensive assessment of the use of healthcare and social services for brain tumor patients at the EOL, as well as information on death places. The study allowed the impact of SPC services to be assessed comprehensively at national level. The strengths of the study are its population-based approach and the inclusion of all adult patients who died from brain tumors in Finland during a year. Limitations of this study include its retrospective design, lack of QOL data and the grouping of late SPC contact (≤ 30 days before death) with no SPC contact. This grouping, based on the understanding that very late SPC is unlikely to significantly impact resource use in the last month of life, allows focus on early SPC integration but may obscure potential differences between late and no SPC. Furthermore, our study design did not allow us to explore the reasons behind the lack of impact of SPC on emergency department visits, which is an area that requires further investigation. Other countries may have different palliative care models, which may affect the comparison between countries.

## Conclusion

Early integration of palliative care is crucial for all brain tumor patients. Our findings demonstrate that it significantly reduces the burden on secondary care, including lowering in-hospital death rates in the last months of life, while enabling more appropriate end-of-life care. This underscores the need for healthcare systems to prioritize the development and expansion of comprehensive SPC services for brain tumor patients.

## Electronic supplementary material

Below is the link to the electronic supplementary material.

Supplementary Material 1

## Data Availability

No datasets were generated or analysed during the current study.

## Abbreviations

EOL: End of life
SPC: Specialist palliative care
QOL: Quality of life
