# Supplementary material for: The impact of palliative care contact on the use of hospital resources at the end of life for brain tumor patients; a nationwide register-based cohort study
Source: J Neurooncol. 2025 Jan 20;172(3):549–56. doi: 10.1007/s11060-025-04939-9 (PMC11968532; doi:10.1007/s11060-025-04939-9)
Supplement: Supplementary file 1 — Supplementary Material 1 [file 11060_2025_4939_MOESM1_ESM.docx]

SUPPLEMENTARY FILE

**Table 1.** **Classification of health care and social care services utilization base**

Data on health care and social service utilization were extracted from three primary sources: the Care Register for Health Care, the Register of Primary Health Care Visits, and the Register of Social Assistance*. These services were then classified into seven distinct categories: secondary health care, primary health care, emergency services, social support, home-based care, hospital at home programs, and specialized palliative care

| Variable | Care Register for Health Care | Register of Primary Health Care visits | Register of Social assistance |
| --- | --- | --- | --- |
| Secondary health care | EA ≠ 98 OR  (EA = 98 AND (PALA ≠ 1 AND YHTEYSTAPA ≠ R80)) |  |  |
| Primary health care | EA = 98 AND (PALA = 1 OR YHTEYSTAPA = R80) | KAYNTI_PALVELUMUOTO ≠ T40, T41, T42, T43 |  |
| Emergency care | PALA = 91 OR  ((KIIREELLISYYS = 5, 6) AND (YHTEYSTAPA = R10, R20, R30, R41, R52, R56 OR R90)) | KAYNTI_KIIREELLISYYS = 1 |  |
| Social services |  |  | PALVELUALA ≠ 7 |
| Home care | PALA = 7 | KAYNTI_PALVELUMUOTO = T40, T41, T42 | PALVELUALA = 7 |
| Hospital at home | Contact linked to specialist palliative care unit (hospital-at-homeunit) | KAYNTI_PALVELUMUOTO = T43  Contact linked to specialist palliative care unit (hospital at home unit) | Contact linked to specialist palliative care unit (hospital at home unit) |
| Specialist palliative care | Contact linked to specialist palliative care unit | Contact linked to specialist palliative care unit | Contact linked to specialist palliative care unit |

EA = Erikoisala (Speciality); YHTEYSTAPA = Yhteystapa (Contact type); PAL= Palveluala (Service branch); KAYNTI_PALVELUMUOTO = Palvelumuoto (Service type); KAYNTI_KIIREELLISYYS = Kiireellisyys (Urgency of care); PALVELUALA = Palveluala (Service branch); KIIREELLISYYS = Kiireellisyys (Urgency of care)

* Register descriptions, The Finnish Institute for Health and Welfare (THL), <https://thl.fi/en/web/thlfi-en/statistics-and-data/data-and-services/register-descriptions>
